# Ultra-Weak Photon Emission from Crown Ethers Exposed to Fenton’s Reagent Fe^2+^-H_2_O_2_

**DOI:** 10.3390/molecules30153282

**Published:** 2025-08-05

**Authors:** Michał Nowak, Krzysztof Sasak, Anna Wlodarczyk, Izabela Grabska-Kobylecka, Agata Sarniak, Dariusz Nowak

**Affiliations:** 1 Radiation Protection, University Hospital No 2, Medical University of Lodz, Zeromskiego 113, 90-549 Lodz, Poland; m.nowak@skwam.lodz.pl; 2 Department of Medical Imaging Techniques, Medical University of Lodz, Lindleya 6, 90-131 Lodz, Poland; krzysztof.sasak@umed.lodz.pl; 3 Department of Sleep Medicine and Metabolic Disorders, Medical University of Lodz, Mazowiecka 6/8, 92-215 Lodz, Poland; anna.wlodarczyk@umed.lodz.pl; 4 Department of Clinical Physiology, Medical University of Lodz, Mazowiecka 6/8, 92-215 Lodz, Poland; izabela.grabska-kobylecka@umed.lodz.pl (I.G.-K.); agata.sarniak@umed.lodz.pl (A.S.)

**Keywords:** ultra-weak photon emission, chemiluminescence, Fenton system, crown ethers, ether bonds, hydroxyl radicals

## Abstract

We hypothesized that compounds containing ether linkages within their backbone structures, when exposed to hydroxyl radicals (•OH), can generate ultra-weak photon emission (UPE) as a result of the formation of triplet excited carbonyl species (^3^R=O*). To evaluate this hypothesis, we investigated the UPE of four compounds, each at a final concentration of 185.2 µmol/L: EGTA (ethylene glycol-bis(β-aminoethyl ether)-N,N,N′,N′-tetraacetic acid), a potent chelator of divalent cations, and three crown ethers—12-crown-4, 15-crown-5, and 18-crown-6—containing two, four, five, and six ether bonds, respectively. •OH was generated using a modified Fenton reagent—92.6 µmol/L Fe^2+^ and 2.6 mmol/L H_2_O_2_. The highest UPE was recorded for the Fe^2+^–EGTA–H_2_O_2_ (2863 ± 158 RLU; relative light units), followed by 18-crown-6, 15-crown-5, and 12-crown-4 (1161 ± 78, 615± 86, and 579 ± 109 RLU, respectively; *p* < 0.05), corresponding to the number of ether groups present. Controls lacking either H_2_O_2_ or Fe^2+^ exhibited no significant light emission compared to the buffer medium. These findings support the hypothesis that ether bonds, when oxidatively attacked by •OH, undergo chemical transformations resulting in the formation of ^3^R=O* species, the decay of which is associated with UPE. In crown ethers exposed to Fe^2+^-H_2_O_2_, the intensity of UPE was correlated with the number of ether bonds in their structure.

## 1. Introduction

### 1.1. Ultra-Weak Photon Emission from Biomolecules

Ultra-weak photon emission (UPE) is a phenomenon observed in living organisms that occurs spontaneously, without the requirement of external excitation or pharmacological stimulation [1,2]. It originates from aerobic metabolic processes, the formation of electronically excited reactive oxygen species (ROS), and oxidative reactions involving lipids, proteins, nucleic acids, and other biomolecules. Oxidative insult to cellular macromolecules initiates a cascade of chemical transformations that result in the formation of various intermediates bearing electronically excited moieties—such as 1,2-dioxetanes, tetroxides, and triplet excited carbonyls (^3^R=O*)—or the generation of singlet oxygen, both of which can serve as sources of spontaneous photon emission [1,2]. As such, UPE has been proposed as a non-invasive biomarker for monitoring oxidative processes and redox homeostasis at the tissue and cellular levels [2,3]. For example, elevated UPE has been documented in porcine skin following chemically induced inflammation via the Fenton reaction (Fe^2+^–H_2_O_2_) [4], and alterations in both intensity and spectral characteristics of skin UPE have been observed in humans with diabetes mellitus, erythropoietic protoporphyria, and exposure to cold stress [1,5]. Among the known contributors to UPE in biological systems, ^3^R=O* species (in addition to singlet oxygen) appear to play a predominant role, particularly in normal and inflamed tissues [4,6]. Emission from ^3^R=O* typically spans the 350–550 nm range [7], which contrasts with the spectral profile of singlet oxygen, characterized by three distinct bands at 1270 nm, 703 nm, and 634 nm [8]. A variety of biological substrates, including oxidatively modified proteins, protein carbonyls, lipid peroxides, peroxyl radicals, and low-molecular-weight compounds (e.g., uric acid, vitamin B12, tryptophan), have been identified as potential precursors of ^3^R=O* [4,6,7]. These excited carbonyl species exhibit short lifespans and undergo rapid radical-like transformations, such as hydrogen abstraction, Norrish-type cleavage, cycloaddition, rearrangement, or polymerization [9]. Consequently, the selective detection of photon emission originating from ^3^R=O* may serve as a powerful tool for assessing oxidative damage to key biomolecular targets such as proteins, amino acids, and lipids in both chemical and biological matrices. Furthermore, attenuation of UPE by exogenous agents has been successfully utilized as an indirect metric for evaluating the antioxidant capacities of dietary polyphenols, vitamins, and other redox-active small molecules [10,11,12].

### 1.2. Ultra-Weak Photon Emission from Fe^2+^–EGTA–H_2_O_2_System: Mechanisms and Possible Application for Determination of Redox Activities of Phytochemicals

Several years ago, we developed a modified Fenton system composed of Fe^2+^, EGTA (ethylene glycol-bis(β-aminoethyl ether)-N,N,N′,N′-tetraacetic acid), and H_2_O_2_ that is capable of emitting ultra-weak photon emission (UPE) under in vitro conditions [12]. By employing a highly sensitive chemiluminometer (AutoLumat Plus LB 953, Berthold Technologies) equipped with a photomultiplier tube operating at 8 °C and detecting photons in the spectral range of 380–630 nm, we demonstrated that, under our experimental conditions, photons originating from singlet oxygen decay do not contribute significantly to the overall UPE signal of the Fe^2+^–EGTA–H_2_O_2_ system. This was verified by the comparative application of H_2_O_2_ and NaOCl, which are known generators of singlet oxygen [13]. Furthermore, we established that this chemiluminescent system is a reliable and sensitive platform for investigating both the antioxidant and pro-oxidant properties of vitamin C and a variety of plant-derived polyphenols [14,15]. EGTA, a potent chelator of divalent metal ions, contains two ether linkages in its molecular structure. We hypothesized that hydroxyl radicals (•OH) generated through the redox reaction of Fe^2+^ with H_2_O_2_ in the presence of EGTA could target and cleave these ether bonds [8,16,17], leading to the formation of organic radicals. In our previous study, we proposed a mechanism of ^3^R=O* formation during exposure of EGTA containing ether bonds to •OH radicals [12]. However, in the light of additional publications and discussions with other scientists, it seems that this explanation contains severe simplifications and mistakes [12]. •OH radicals react with ether bonds via hydrogen atom abstraction mainly at the carbon atom adjacent to the oxygen atom [18]. This leads to the breakdown of the ether bond and formation of alpha-alkoxy radicals and water [18]. This mechanism is one of those responsible for oxidative degradation of lignin in the ether bonds [19,20]. Alpha–alkoxy radicals can convert into carbon-centered radicals via β-scission and hydrogen atom transfer [21,22]. They can react with O_2_ dissolved in the reaction milieu and form peroxyl radicals [23,24] which can convert into ^3^R=O* [25] (e.g., via recombination of two peroxyl radicals, formation of tetroxide and its decomposition) with subsequent photon emission [12]. The yield of ^3^R=O* is very low; therefore, the intensity of light emission related to triplet excited carbonyl decay is a thousand times lower than that originated from singlet oxygen, which is also formed in the aforementioned processes [26]. Moreover, one can expect that UPE derived from ^3^R=O* may depend on the partial pressure of O_2_ dissolved in the reaction milieu. This mechanistic proposal was further supported by negative control experiments involving two additional modified Fenton systems: Fe^2+^–citrate–H_2_O_2_ and Fe^2+^–EDTA–H_2_O_2_. Although both citrate and EDTA are effective chelators of Fe^2+^ ions, neither compound contains ether linkages within their molecular frameworks. Consequently, neither system exhibits measurable UPE, corroborating the essential role of ether bonds in photon generation under these reaction conditions [12].

### 1.3. Study Aims

In the present study, we aimed to validate the hypothesis that photon emission (UPE) observed in the Fe^2+^–EGTA–H_2_O_2_ system arises from the oxidative degradation of ether bonds by hydroxyl radicals (•OH). To this end, we designed a series of experiments in which structurally distinct compounds containing ether linkages were exposed to •OH radicals generated via the classical Fenton reaction (Fe^2+^–H_2_O_2_), followed by measurement of UPE. For this purpose, we selected three commercially available water-soluble crown ethers—12-Crown-4, 15-Crown-5, and 18-Crown-6—comprising macrocyclic rings with four, five, and six ether groups, respectively. Upon exposure to •OH radicals, all three crown ethers exhibited measurable UPE. This observation provides compelling evidence that ether bonds represent a critical structural motif susceptible to •OH-mediated cleavage, ultimately contributing to the generation of triplet excited carbonyl species and the resultant ultra-weak photon emission.

## 2. Results and Discussion

**Figure 1:**
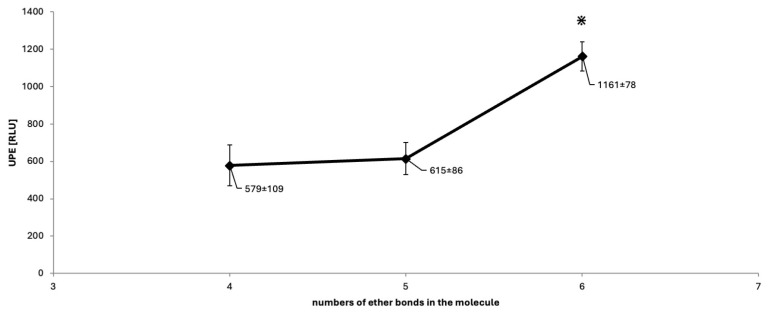
Relationship between UPE (mean ± SD) and the number of ether bonds in the backbone structure of the studied crown ethers. 12-Crown-4, 15-Crown-5, and 18-Crown-6 contain four, five, and six ether bonds, respectively. UPE is expressed in relative light units (RLU). The final concentrations of Fe^2+^, crown ether, and H_2_O_2_ in the reaction mixture were 92.6 µmol/L, 185.2 µmol/L, and 2.6 mmol/L, respectively. * *p* < 0.05 vs. 12-Crown-4 and 15-Crown-5.

Table 1 presents the chemical structures of the studied crown ethers (12-Crown-4, 15-Crown-5, and 18-Crown-6) alongside the reference compound, EGTA, as well as the levels of ultra-weak photon emission (UPE) generated following their exposure to the Fe^2+^–H_2_O_2_ Fenton reagent. Although all crown ethers possess a greater number of ether bonds than EGTA, the corresponding systems—12-Crown-4–Fe^2+^–H_2_O_2_, 15-Crown-5–Fe^2+^–H_2_O_2_, and 18-Crown-6–Fe^2+^–H_2_O_2_—produced significantly lower UPE, by factors of 4.9, 4.7, and 2.5, respectively (*p* < 0.05), when compared to the Fe^2+^–EGTA–H_2_O_2_ system (2863 ± 158 RLU; median: 2853; IQR: 96). Crown ethers are known to undergo reactions with •OH radicals in aqueous environments, with reaction rates positively correlated with the number of hydrogen atoms present in the molecule, suggesting that hydrogen atom abstraction is the predominant mechanism of •OH-induced degradation in these compounds [27]. Consequently, the cleavage of ether bonds and the formation of triplet excited carbonyls (^3^R=O*) leading to photon emission appear to be less efficient in crown ethers than in EGTA, accounting for the lower UPE observed in their respective systems. Furthermore, EGTA acts as a potent chelator of Fe^2+^ ions, facilitating the localized generation of •OH radicals in close proximity to its ether bonds, thereby increasing the likelihood of ^3^R=O* formation. In contrast, while crown ethers are also capable of coordinating various divalent cations [28,29], including potentially Fe^2+^, this chelation mechanism does not appear to play a significant role under the conditions of the present study. Figure 1 illustrates the relationship between the number of ether bonds in the backbone of crown ethers and the corresponding UPE. Although compounds with four and five ether bonds (12-Crown-4 and 15-Crown-5) exhibited similar levels of light emission, both were significantly lower (*p* < 0.05) than that observed for 18-Crown-6 (1161 ± 78 RLU; median: 1143; IQR: 72), suggesting that a higher number of ether bonds enhances the likelihood of •OH-induced cleavage and, consequently, UPE intensity. It is also noteworthy that •OH radicals and superoxide radicals (also generated in Fenton’s reagent [30] can react with ^3^R=O* species [31,32,33], which can perpetuate further ether bond cleavage in the crown ether structures as well as photon emission after triplet excited state decay. The results of experiments with crown ethers exposed to Fenton reagent clearly show that oxidative attack on ether bonds may initiate a chain of redox reactions involving a variety of radicals (alkoxy radicals, carbon-centered radicals, peroxyl radicals), leading to the formation of ^3^R=O* and subsequent emission of photons (UPE). This is the first report (to the best of our knowledge) on UPE emission from crown ethers exposed to Fe^2+^-H_2_O_2_, and due to the simplicity of the system (only three components), it may be used for the determination of redox properties of various compounds, including phytochemicals (e.g., polyphenols). Moreover, interpretation of the results may be much easier than in the case of Fe^2+^-EGTA-H_2_O_2_ because the chelating activity of crown ethers is many times lower than that of EGTA. The luminometer software enables only measurement of total light emission over the fixed time. There was no way to monitor changes in the intensity of UPE over the time. Therefore, we were not able to analyze kinetics of photon emission and compare them between the studied systems of Fe^2+^-EGTA-H_2_O_2_, 12-crown-4–Fe^2+^-H_2_O_2_, 15-crown-5–Fe^2+^-H_2_O_2_, and 18-crown-6—Fe^2+^-H_2_O_2_. This could be recognized as quite a serious limitation of our study. Table 2 summarizes the light emission profiles of the control systems. Photon emission from incomplete reaction systems (e.g., crown ether–H_2_O_2_ and crown ether–Fe^2+^) remained low and comparable to that of the medium alone. The UPE generated by the 18-crown-6–Fe^2+^–H_2_O_2_ system demonstrated low inter-assay variability, with a standard deviation of approximately 7% of the mean value (Figure 1), indicating the potential utility of 18-crown-6 as a stable and reproducible UPE-generating system for evaluating the antioxidant capacity of various water-soluble phytochemicals and synthetic compounds. These findings align with our previous observations, where control systems consistently exhibited low and stable baseline UPE [12,14].

## 3. Directions for Future Research

We found that the Fe^2+^–Crown ether–H_2_O_2_ system is an efficient generator of •OH-dependent ultra-weak photon emission (UPE). The primary question to be addressed in future studies is the potential applicability of this system for evaluating the redox properties—both pro- and antioxidant—of phytochemicals, particularly plant polyphenols and other redox-active compounds soluble in water. To resolve this issue, further research is required to optimize the reaction conditions, including the pH of the medium, to maximize the UPE signal-to-noise ratio, the selection of the most effective crown ether, and the concentrations of system components. Additionally, preliminary experiments with selected polyphenols should be conducted. The use of alternative metal cations (e.g., Cu^2+^, Cr^3+^, or Co^2+^) in place of Fe^2+^ also warrants investigation in the context of the proposed future research directions.

## 4. Material and Methods

### 4.1. Chemicals and Solutions

All chemicals used were of analytical grade. Sodium phosphate monobasic monohydrate (NaH_2_PO_4_ × H_2_O), sodium phosphate dibasic heptahydrate (Na_2_HPO_4_ × 7H_2_O), iron(II) sulfate heptahydrate (FeSO_4_ × 7H_2_O), sodium hydroxide (NaOH), ethylene glycol-bis(β-aminoethyl ether)-N,N,N′,N′-tetraacetic acid (EGTA), and the crown ethers 12-Crown-4, 15-Crown-5, and 18-Crown-6 were obtained from Sigma-Aldrich Chemicals (St. Louis, MO, USA). A 30% (*w*/*w*) hydrogen peroxide (H_2_O_2_) solution was purchased from Chempur (Piekary Śląskie, Poland). Sterile, deionized, pyrogen-free water (freshly prepared; resistance > 18 MΩ·cm, HPLC H_2_O Purification System, USF Elga, Buckinghamshire, UK) was used throughout the study. Working solutions of FeSO_4_ (5 mmol/L), EGTA (10 mmol/L), and H_2_O_2_ (28 mmol/L), as well as a 10 mmol/L phosphate buffer (PB, pH 6.6), were prepared according to previously described protocols [13].

### 4.2. Preparation of Aqueous Solutions of Crown Ethers

According to the PubChem database, the following physicochemical properties were reported for the studied crown ethers: 12-Crown-4 is miscible with water in all proportions, with a melting point of 16 °C and a liquid density of 1.089 g/mL; 15-Crown-5 is also completely miscible with water, remains liquid at room temperature, and has a density of 1.113 g/mL at 20 °C; 18-Crown-6 exhibits a water solubility of 75 g/L, a melting point of 37–40 °C, and a density of 1.237 g/mL. Working solutions of the crown ethers were prepared in 10 mmol/L phosphate buffer (PB, pH 6.6) as follows: 16.1 µL of 12-Crown-4 was added to 9980 µL of PB; 19.7 µL of 15-Crown-5 was added to 9960 µL of PB; and 18-Crown-6 was first incubated at 41 °C for 30 min to ensure liquefaction, after which 21.26 µL of the compound was mixed with 9959 µL of PB. The final concentration of each crown ether in the respective working solutions was 9.95 mmol/L.

### 4.3. Light-Emitting Systems and Measurements of Ultra-Weak Photon Emission

In our previous studies, the modified Fenton system composed of 92.6 µmol/L Fe^2+^, 185.2 µmol/L EGTA, and 2.6 mmol/L H_2_O_2_ in 10 mmol/L phosphate buffer (PB, pH 6.6) demonstrated distinct ultra-weak photon emission (UPE) [12,13]. The emission process was shown to depend on the oxidative cleavage of the two ether bonds present in the backbone structure of EGTA [12]. Therefore, in the present study, the Fenton systems involving crown ethers were prepared using the same concentrations: 92.6 µmol/L Fe^2+^, 185.2 µmol/L crown ether, and 2.6 mmol/L H_2_O_2_ in 10 mmol/L PB (pH 6.6). The Fe^2+^–EGTA–H_2_O_2_ system was used as the reference UPE generator. The experimental protocol, including all control conditions, is detailed in Table 3. Total light emission was measured using a multitube luminometer (AutoLumat Plus LB 953, Berthold, Germany), equipped with a Peltier-cooled photon counter (spectral range: 380–630 nm), maintained at 8 °C to ensure high sensitivity and low background signal. In brief, 20 µL of a 9.95 mmol/L aqueous solution of crown ether (12-Crown-4, 15-Crown-5, or 18-Crown-6) was added to a Lumi Vial Tube (5 mL, 12 × 75 mm; Berthold Technologies, Bad Wildbad, Germany) containing 940 µL of phosphate buffer (pH 6.6). Next, 20 µL of a 5 mmol/L FeSO_4_ solution was added. After gentle mixing, the tube was placed in the luminometer and incubated in the dark at 37 °C for 10 min. Subsequently, 100 µL of a 28 mmol/L H_2_O_2_ solution was automatically injected, and UPE was recorded over 120 s, expressed in relative light units (RLU). Control conditions included incomplete systems consisting of Fe^2+^–H_2_O_2_, crown ether–H_2_O_2_, Fe^2+^–crown ether, H_2_O_2_ alone, and buffer alone (PB–H_2_O), as outlined in Table 3. UPE values for the Fe^2+^–crown ether–H_2_O_2_ and Fe^2+^–EGTA–H_2_O_2_ systems were calculated using the following formulas: UPE = (light emission from Fe^2+^–crown ether–H_2_O_2_)−(light emission from buffer alone) and UPE = (light emission from Fe^2+^–EGTA–H_2_O_2_)−(light emission from buffer alone). All relevant experimental conditions (samples 1–6; Table 3) were analyzed concurrently within each experimental run to minimize inter-assay variability. Each experiment was independently repeated ten times.

### 4.4. Statistical Analysis

UPE generated by EGTA and crown ethers upon exposure to Fe^2+^–H_2_O_2_, along with light emission from the corresponding control systems, is presented as the mean (standard deviation, SD) and median with interquartile range (IQR). Statistical comparisons between UPE values of the Fe^2+^–EGTA–H_2_O_2_ system and those of Fe^2+^–crown ether–H_2_O_2_ systems, as well as control photon signals, were conducted using either the independent-samples (unpaired) *t*-test or the Mann–Whitney U test, depending on data distribution as determined by the Kolmogorov–Smirnov–Lilliefors test. Homogeneity of variances was assessed using the Brown–Forsythe test; if unequal variances were detected, Welch’s *t*-test was employed in place of the standard *t*-test. A *p*-value < 0.05 was considered indicative of statistical significance.

## 5. Conclusions

Crown ethers exposed to hydroxyl radicals (•OH) generated via the Fenton reaction (Fe^2+^–H_2_O_2_) exhibited measurable ultra-weak photon emission (UPE). Notably, compounds with a higher number of ether bonds in their backbone structure produced greater photon emission upon •OH exposure. These findings provide further evidence supporting the hypothesis that ether bonds and their oxidative transformation play a critical role in the generation of triplet excited carbonyl species and the associated light emission. Further investigations are warranted to determine whether UPE-generating systems comprising crown ethers and Fenton’s reagent may serve as reliable tools for assessing the pro- or antioxidant activity of various compounds, including naturally occurring phytochemicals.

## Figures and Tables

**Table 1:** Chemical structures of EGTA and the investigated crown ethers (12-Crown-4, 15-Crown-5, and 18-Crown-6), along with the corresponding levels of ultra-weak photon emission (UPE) following exposure to hydroxyl radicals (•OH) generated by the Fe^2+^–H_2_O_2_ Fenton reagent.

Compound	Chemical Structure with Marked in Red Ether Bonds	Number of Ether Bonds (R−O−R’) in the Backbone Structure	UPE After Exposure to Fe^2+^-H_2_O_2_ [RLU]
EGTA	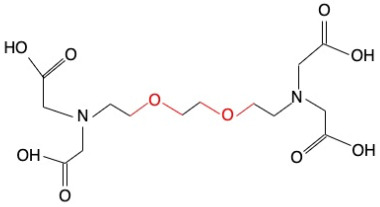	2	2863 ± 58 (2853; 96) †
12-Crown-4	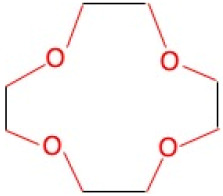	4	579 ± 109 (589; 76)
15-Crown-5	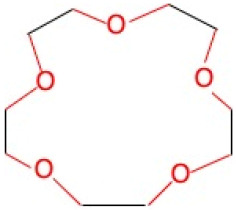	5	615 ± 86 (623; 67)
18-Crown-6	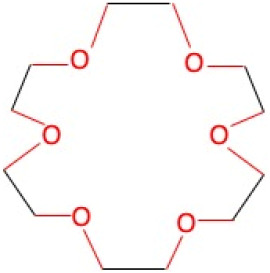	6	1161 ± 78 (1143; 72) *

EGTA refers to ethylene glycol-bis(β-aminoethyl ether)-N,N,N′,N′-tetraacetic acid. Crown ethers are macrocyclic compounds composed of repeating ether units (R−O−R′) arranged in a ring structure. UPE (ultra-weak photon emission) was calculated by subtracting the baseline photon emission of the medium alone from the total light emission measured in systems containing 92.6 µmol/L Fe^2+^, 185.2 µmol/L of either a crown ether or EGTA, and 2.6 mmol/L H_2_O_2_. UPE values are expressed in relative light units (RLU) and presented as mean (standard deviation) and median (interquartile range, IQR). *—significantly different compared to the UPE of the 12-Crown-4–Fe^2+^–H_2_O_2_ and 15-Crown-5–Fe^2+^–H_2_O_2_ systems, *p* < 0.05; †—significantly different compared to the UPE of all tested crown ether systems, *p* < 0.05.

**Table 2:** Light emission from control systems involved in experiments with studied crown ethers: 12-crown-4, 15-crown-5, and 18-crown-6.

Experiment	Light Emission from Control Systems [RLU]				
	Medium Alone	H_2_O_2_ Alone	Crown Ether–Fe^2+^	Crown Ether–H_2_O_2_	Fe^2+^-H_2_O_2_
12–Crown-4	607 ± 64 (636;115)	585 ± 76 (625;132)	596 ± 64 (631;108)	568 ± 76 (603;145)	637 ± 69 (650;133)
15-Crown-5	578 ± 72 (578;130)	682 ± 55 (682;95)	575 ± 75 (520;132)	549 ± 70 (549;125)	628 ± 63 (630;98)
18–Crown-6	662 ± 20 (662;13)	659 ± 27 (658;43)	655 ± 32 (658;39)	662 ± 29 (670;51)	822 ± 52 (822;50)

Each experiment with a given crown ether included five control conditions: medium alone, H_2_O_2_ alone, crown ether + Fe^2+^, crown ether + H_2_O_2_, and Fe^2+^ + H_2_O_2_, all conducted simultaneously. UPE was measured as the difference in light emission between each control system and the medium alone, expressed in relative light units (RLU) and presented as mean (SD) and median (interquartile range—IQR). The final concentrations of crown ether, Fe^2+^, and H_2_O_2_ in the control systems were 185.2 µmol/L, 92.6 µmol/L, and 2.6 mmol/L, respectively. Light emission from all control systems was low and comparable to the background level.

**Table 3:** Design of experiments on light emission (UPE) from crown ethers exposed to Fe^2+^-H_2_O_2_ system.

Experiment Number	Sample	Volumes of Working Solutions Added to Luminometer Tube (µL)				
		A	B	C	D	E
		PB (pH = 6.6)	Crown Ether	Fe^2+^	H_2_O_2_	H_2_O
1	Complete system Fe^2+^-Crown ether-H_2_O_2_	940	20	20	100	-
2	Incomplete system Fe^2+^-H_2_O_2_	960	-	20	100	-
3	Incomplete system Crown ether-H_2_O_2_	960	20	-	100	-
4	H_2_O_2_ alone	980	-	-	100	-
Additional controls						
5	Crown ether-Fe^2+^	940	20	20	-	100
6	Medium alone	980	-	-	-	100

Working solutions were mixed in the following order: A—10 mmol/L phosphate buffer (PB, pH = 7.4); B—9.95 mmol/L aqueous solution of a crown ether (12-Crown-4, 15-Crown-5, or 18-Crown-6); C—5 mmol/L aqueous solution of FeSO_4_. After gentle mixing, the tube was placed into the luminometer carousel and incubated in the dark at 37 °C for 10 min. Subsequently, 100 µL of either 28 mmol/L H_2_O_2_ (D) or water (E) was automatically injected using a dispenser, and total light emission was measured over a 2 min period. In specific experiments, an equivalent concentration of EGTA was used in place of the crown ether solution.

## Data Availability

The data supporting the findings of this study are available upon request from the corresponding author.
